# Effects of prolonged embryo cryopreservation after fertility treatment in Denmark

**DOI:** 10.1530/RAF-25-0185

**Published:** 2026-06-18

**Authors:** Emilie Høyer Preisler, Morten Rønn Petersen, Marie Louise Grøndahl, Anette Gabrielsen, Stine Ravn Hansen, Tilde Veng Eskildsen, Betina Boel Povlsen, Bettina Troest, Dea Frøding Skipper, Emilie Steentoft Dahlberg, Henriette Svarre Nielsen, Nina la Cour Freiesleben, Anne Zedeler

**Affiliations:** ^1^ The Fertility Clinic, Department of Obstetrics and Gynaecology, Copenhagen University Hospital Hvidovre, Hvidovre, Denmark; ^2^ The Fertility Clinic, Copenhagen University Hospital Rigshospitalet, Copenhagen, Denmark; ^3^ The Fertility Clinic, Department of Obstetrics and Gynaecology, Copenhagen University Hospital Herlev, Herlev, Denmark; ^4^ Department of Clinical Medicine, Aarhus University, Denmark and The Fertility Clinic Horsens, Horsens Regional Hospital, Aarhus, Denmark; ^5^ Department of Obstetrics and Gynaecology, The Fertility Clinic, Zealand University Hospital Koege, Koege, Denmark; ^6^ The Fertility Clinic, Odense University Hospital, Odense, Denmark; ^7^ University Clinic for Fertility, Central Denmark Region, Skive and Department of Clinical Medicine, Aarhus University, Aarhus, Denmark; ^8^ Fertility Unit, Department of Obstetrics and Gynaecology, Aalborg University Hospital, Aalborg, Denmark; ^9^ Department of Obstetrics and Gynaecology, Copenhagen University Hospital Hvidovre, Hvidovre, Denmark; ^10^ Department of Clinical Medicine, University of Copenhagen, Copenhagen, Denmark

**Keywords:** embryo cryopreservation, storage time, frozen embryo transfer, ongoing pregnancy rate, ART legislation

## Abstract

**Abstract:**

This retrospective cohort study examined the effects of the Danish legislative amendment extending the permitted duration of embryo cryopreservation from five years until the woman reaches 46 years on frozen embryo transfer (FET) during assisted reproductive technology. The number of embryos discarded across the eight public fertility clinics due to the five-year storage limit was quantified between January 1, 2017, and December 31, 2019, with an average annual destruction of 1,399 embryos. As of March 2024, 29,591 embryos were cryopreserved in the public fertility clinics. FET cycles from the public clinics between October 2020 and March 2024 were included in the outcome analysis (*n* = 14,677), of which 1.9% (*n* = 284) involved embryos cryopreserved >5 years. Ongoing pregnancy rate (OPR) and live birth rate (LBR) were assessed by parity and cryopreservation duration (≤5 years vs >5 years). OPR and LBR increased significantly with parity (36.7 and 31.3% for first, 42.6 and 35.6% for second, and 48.8 and 39.0% for third/fourth child), although the increase in LBR for first vs third/fourth child was not statistically significant. OPR and LBR in FET cycles with embryos cryopreserved >5 years were not significantly different from cycles with embryos cryopreserved ≤5 years (OPR: 36.4 vs 38.0%; LBR: 30.9 vs 32.2%). During the study period, 84 children were born from embryos cryopreserved >5 years that previously would have been destroyed. The findings suggest that the amendment has enabled patients to use surplus embryos for family expansion and prevented the disposal of many good embryos.

**Lay summary:**

In fertility treatment, cryopreservation is the process of freezing fertilized eggs called embryos. Embryos can be warmed and transferred to the uterus in a future treatment cycle, giving patients another chance at pregnancy without repeating egg retrieval and fertilization. In Denmark, a recent legislative amendment extended the storage limit for embryos from five years to the woman’s 46th birthday. This study investigated how this amendment affected the use of cryopreserved embryos as well as pregnancy and live birth outcomes after FET. Few transfers involved embryos cryopreserved over five years. Pregnancy and live birth rates were similar whether embryos had been frozen under or over five years, and 84 children were born from embryos stored over five years that previously would have been destroyed. The findings suggest that the amendment has enabled patients to use embryos from previous treatments for family expansion and reduced unnecessary destruction of good embryos.

## Introduction

Frozen embryo transfer (FET) is an integral component of assisted reproductive technology (ART), and its use in Denmark has increased substantially since embryo cryopreservation was legalized in 1992. In 2025, Danish fertility clinics performed 10,047 FET treatments compared with 1,341 FET treatments in 2001 (Danish Fertility Society 2025, 2026). This rise in FET reflects major developments in ART practice. In earlier years, multiple embryos were often transferred in the fresh cycle to improve pregnancy rates, significantly increasing the risk of multiple gestations (Andersen 2021) and associated complications, such as low birth weight, preterm delivery, and adverse obstetric outcomes (Pinborg *et al.* 2005, Martin *et al.* 2017). With the integration of embryo culture to the blastocyst stage combined with cryopreservation, elective single embryo transfer (eSET) has gradually become the standard and recommended clinical practice in many countries (ESHRE Guideline Group on the Number of Embryos to Transfer *et al.* 2024). In Denmark, this transition has been particularly evident with the proportion of eSET increasing from 32.6% of transfers in 2005 to 99.1% in 2025 (Nyboe Andersen *et al.* 2009, Danish Fertility Society 2025, 2026). As a result, multiple gestations and the associated risks have declined, while surplus embryos can be cryopreserved for future reproductive use (Andersen 2021, Canosa *et al.* 2022).

In addition to reducing multiple gestations, FET has been associated with better clinical outcomes in terms of ongoing pregnancy rates (OPRs) compared with fresh embryo transfer (Stormlund *et al.* 2017). An important technological advancement contributing to improved FET outcomes has been the transition from slow freezing to vitrification, enhancing post-warming survival rates of embryos cultured to the blastocyst stage (Loutradi *et al.* 2008). Other clinical advantages of FET include avoidance of ovarian hyperstimulation syndrome (OHSS) through agonist trigger and a freeze-all strategy, postponed embryo transfer for optimal timing of the endometrium, and the potential of fewer oocyte retrievals in each patient (Canosa *et al.* 2022).

Alongside these clinical developments, the legal framework governing embryo cryopreservation in Denmark has evolved. Initially, embryos could be stored for up to 12 months, a limit later extended to 24 months in 1997, and to five years in 2006 (*Lov om ændring af lov om assisteret reproduktion i forbindelse med behandling, diagnostik og forskning m.v.* 2006) (Act amending the Danish Act on Assisted Reproduction in connection with treatment, diagnostics, and research, etc. (2006)). On January 1, 2021, a legislative amendment prompted by a citizen’s proposal was implemented, extending the permitted cryopreservation duration of embryos by removing the five-year storage limit and instead allowing embryos to be cryopreserved until the woman turns 46 years (*Lov om ændring af lov om assisteret reproduktion i forbindelse med behandling, diagnostik og forskning m.v.* 2021) (Act amending the Danish Act on Assisted Reproduction in connection with treatment, diagnostics, and research, etc. (2021)). Danish law also requires that cryopreserved embryos be destroyed under certain circumstances, such as relationship dissolution, the death of a partner without written consent for posthumous use, or at the request of one of the partners involved (*Lov om ændring af lov om assisteret reproduktion i forbindelse med behandling, diagnostik og forskning m.v.* 2019) (Act amending the Danish Act on Assisted Reproduction in connection with treatment, diagnostics, and research, etc. (2019)).

### Study purpose

The aim of this study was to investigate the effect of the legislative amendment by evaluating FET cycles in Denmark following the implementation of the legislation extending the maximum duration of embryo cryopreservation. The study assesses clinical outcomes of FET cycles by duration of cryopreservation (≤5 years vs >5 years) and parity and quantifies the number of embryos destroyed due to the previous five-year storage limit and the number of cryopreserved embryos stored at the public fertility clinics as of March 1, 2024.

## Materials and methods

There are eight public fertility clinics in Denmark, all of which perform embryo cryopreservation for FET. This retrospective cohort study was based on registry data on FET cycles collected from all eight clinics – Copenhagen University Hospital Rigshospitalet, Copenhagen University Hospital Hvidovre, Copenhagen University Hospital Herlev, Zealand University Hospital Koege, Odense University Hospital, Regional Hospital Skive, Regional Hospital Horsens, and Aalborg University Hospital. Each of the clinics was asked to provide a) the number of cryopreserved embryos as of March 1, 2024, b) the number of embryos discarded after five-year storage in the period from January 1, 2017, to December 31, 2019, before the new legislation on prolonged cryopreservation. This period was chosen as most clinics had reduced activity between 2020 and 2021 due to the COVID-19 pandemic, and therefore this time period was not representative, and c) data on all warmed embryos from October 2020 until March 2024.

### Inclusion and exclusion criteria

FET and FET-PGT.A cycles with at least one vitrified–warmed cleavage-stage embryo or blastocyst between October 1, 2020, and March 1, 2024, were included. Cycles with missing data on oocyte pickup (OPU) or embryo warming date were excluded because the duration of embryo cryopreservation could not be calculated.

### Patient groups

Patients were grouped into four categories: heterosexual couples using partner’s sperm, heterosexual couples using donor sperm, single women or lesbian couples using donor sperm, and unknown relationship status.

### Duration of cryopreservation

The duration of cryopreservation for the embryos in each cycle was calculated as the difference between the embryo warming date and the OPU date.

### The woman’s age at the OPU day

The dataset did not provide the women’s birth dates, and therefore, the woman’s age at OPU was estimated as a calculated value by rounding the number of years the embryos had been stored down to the nearest whole number and subtracting the number of years from the woman’s age at embryo warming. For example, if a woman was 35 years old at warming, and the embryos had been stored for three years and seven months, the storage time was rounded down to three years, resulting in an OPU age of 32 years.

### FET protocol and freezing/warming protocol

FET protocols followed local practice at each clinic and included 7,220 (49.2%) modified natural cycle FET (mNC-FET), 5,284 (36.0%) hormone-substituted cycle FET, and 670 (4.6%) stimulated cycle FET. FET protocol was missing for 1,503 (10.2%) cycles.

Both embryos and blastocysts were cryopreserved by vitrification and subsequent warming. Vitrification and warming protocols followed local practice at each clinic and are specified in Supplementary Table 1 (see section on Supplementary materials given at the end of the article).

### Cycle outcome measures

Ongoing pregnancy, live birth, missed abortion, induced abortion, still birth, and lost to follow-up were quantified for all FET cycles and FET cycles with embryos cryopreserved >5 years. The primary clinical outcome of this study was ongoing pregnancy, defined as the presence of an intrauterine pregnancy with a fetal heartbeat upon a transvaginal ultrasound scan in gestational week 7–8. OPR was calculated as the number of ongoing pregnancies divided by the number of cycles with transfer of at least one embryo. Live birth rate (LBR) was determined as the number of FET cycles with a live birth among cycles with transfer of at least one embryo.

The exact post-warming survival rate of vitrified embryos could not be determined because the dataset did not include embryo viability status following warming. Instead, post-warming survival rates were calculated as the number of transferred embryos divided by the number of embryos warmed. Implantation rate was defined as the number of cycles with an ongoing pregnancy divided by the total number of embryos transferred.

### Statistical analysis

All statistical analyses were performed using RStudio (v4.5.0.). To evaluate differences in OPR and LBR across FET cycles stratified by parity (first vs second and first vs third/fourth child) and by duration of embryo cryopreservation (≤5 years vs >5 years), a logistic mixed-effects model was applied. The model included a random effect for uxor ID to account for repeated measures, as some patients had more than one FET cycle and were represented in multiple parity groups. Odds ratios (ORs) with corresponding 95% confidence intervals (CIs) were derived from the model. The null hypothesis stated that no differences existed in OPR or LBR between parity groups or durations of cryopreservation. A *P*-value < 0.05 was considered statistically significant.

## Results

### Cryopreserved embryos in storage and discarded embryos because of five-year storage law

A total of 29,591 cryopreserved embryos (28,745 blastocyst stage and 846 cleavage stage) were in storage for 9,788 patients as of March 1, 2024, in the public fertility clinics in Denmark. Between January 1, 2017, and December 31, 2019, before the new legislation allowing prolonged cryopreservation took effect, 4,196 embryos were destroyed from 1,619 patients because they reached the maximum storage period of five years, corresponding to an average of 1,399 embryos from 540 patients destroyed annually.

### FET between October 2020 and March 2024

#### Cycle characteristics

**Figure 1:**
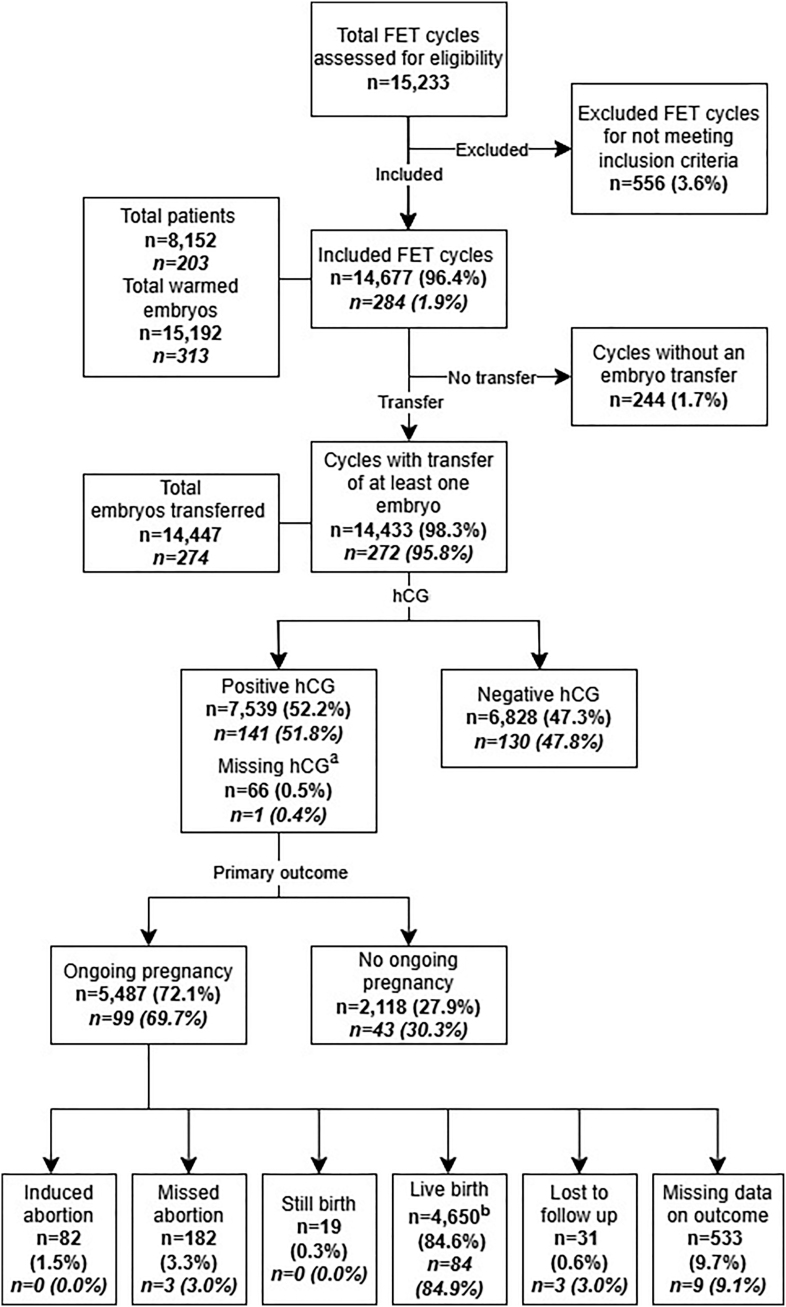
Study flow chart. *n* represents all FET cycles; *n* represents FET cycles with embryos cryopreserved >5 years; ^a^Of the 66 cycles with missing hCG, 8 cycles had an ongoing pregnancy; ^b^10 cycles with a live birth were registered without an ongoing pregnancy.

As seen in the study flow chart in Fig. 1, a total of 14,677 FET cycles with at least one warmed embryo were included for 8,152 patients during the study period between October 2020 and March 2024, while 556 cycles were excluded because they did not meet the inclusion criteria.

Overall, 14,433 cycles included embryo transfer (14,419 cycles were single embryo transfer (SET) and 14 cycles were double embryo transfer (DET)). Among all cycles, 284 (1.9%) included embryos that were cryopreserved >5 years. Of these, 272 were cycles with transfer (270 were SET and two were DET). The longest storage period for a transferred embryo was nine years and five months.

Of all transferred embryos, 99.1% (14,299/14,433) were blastocysts and 0.9% (134/14,433) were cleavage-stage embryos. Among blastocysts (*n* = 14,299), 86.8% (*n* = 12,413) had a Gardner score ≥3BB, 6.0% (*n* = 864) had a score <3BB, and 7.2% (*n* = 1,022) had missing Gardner scores at transfer. The overall positive hCG rate per transfer was 52.2% (*n* = 7,539), the OPR was 38.0% (*n* = 5,487), and the LBR was 32.2% (*n* = 4,650). There were missing data on live birth outcome for 533 cycles (3.6%). The implantation rate was 38.0% (5,487/14,447).

For FET cycles with embryos cryopreserved >5 years, 96.0% (261/272) of transferred embryos were blastocysts and 4.0% (11/272) were cleavage-stage embryos. The positive hCG rate per transfer was 51.8% (*n* = 141), the OPR was 36.4% (*n* = 99), and the LBR was 30.9% (*n* = 84). There were missing data on live birth outcome for nine cycles with embryos cryopreserved >5 years (3.2%). The implantation rate was 36.1% (99/274).

For all embryos, the post-warming survival rate was 95.1% (14,447/15,192), ranging from 90.1 to 97.2% between clinics. For embryos cryopreserved >5 years, the post-warming survival rate was 87.5% (274/313), ranging from 73.2 to 100.0% between clinics.

#### Population characteristics

Table 1 presents the distribution of the 8,152 included patients across the four patient groups and the distribution of FET cycles by parity for all cycles and cycles with embryos cryopreserved >5 years. A subset of 3,575 patients underwent multiple (range: 2–16) FET cycles, and 203 patients had FET cycles with embryos cryopreserved >5 years.

**Table 1:** Patient groups by relationship status and distribution of FET cycles by parity for all patients undergoing at least one FET warm and patients with minimum one FET warm with embryos cryopreserved >5 years.

	Patients		Cycles	
	All	With at least one FET	All*	With EC > 5 years
Heterosexual couples using partner’s sperm	85.6% (6,978/8,152)	87.2% (177/203)		
Heterosexual couples using donor sperm	2.4% (199/8,152)	1.0% (2/203)		
Single women and lesbian couples using donor sperm	11.1% (907/8,152)	10.0% (20/203)		
Unknown relationship status	0.8% (68/8,152)	2.0% (4/203)		
FET for first child			78.6% (11,538/14,677)	22.5% (64/284)
FET for second child			20.5% (3,014/14,677)	56.0% (159/284)
FET for third/fourth child			0.9% (125/14,677)	21.5% (61/284)

* Includes all cycles with at least one warmed embryo.

FET, frozen embryo transfer; EC, embryos cryopreserved.

As seen in Table 1, 78.6% (*n* = 11,538) of all FET cycles were performed in patients receiving treatment for the first child, 20.5% (*n* = 3,014) for the second child, and 0.9% (*n* = 125) for the third/fourth child. For FET cycles with embryos cryopreserved >5 years, 22.5% (*n* = 64) of cycles were performed in patients receiving treatment for the first child, 56.0% (*n* = 159) for the second child, and 21.5% (*n* = 61) for the third/fourth child. Table 1 also displays the distribution of patients’ relationship status for all FET cycles and patients with at least one FET warm with embryos cryopreserved >5 years. Heterosexual couples using partner’s sperm comprised 85.6% (*n* = 6,978) of all patients and 87.2% (*n* = 177) of patients with embryos cryopreserved >5 years.

#### Age distribution at OPU and embryo warming for all patient groups

Table 2 displays the age distribution among all patients at OPU and embryo warming for all cycles with minimum one FET warm and cycles with minimum one FET warm with embryos cryopreserved >5 years. Patients were categorized into four age-groups: ≤34 years, 35–39 years, 40–42 years, and ≥43 years. Additional details on the age distribution at OPU and embryo warming across the different patient groups are provided in Supplementary Table 2.

**Table 2:** Age distribution at OPU and embryo warming for all patients undergoing at least one FET warm and patients with minimum one FET warm with embryos cryopreserved >5 years. Uxor ages from all FET cycles (*n* = 14,677) and from FET cycles with embryos cryopreserved >5 years (*n* = 284) are included. Patients who had multiple cycles with warm are therefore represented more than once.

	Duration of cryopreservation			
	≤34 years	35–39 years	40–42 years	≥43 years
All FET cycles				
Age at OPU	65.8% (9,656/14,677)	26.5% (3,893/14,677)	7.6% (1,119/14,677)	0.1% (9/14,677)
Age at warm	59.7% (8,766/14,677)	30.0% (4,405/14,677)	9.4% (1,377/14,677)	0.9% (129/14,677)
FET cycles (>5 years)				
Age at OPU	78.5% (223/284)	18.3% (52/284)	3.2% (9/284)	-
Age at warm	30.3% (86/284)	46.1% (131/284)	12.4% (36/284)	10.9% (31/284)

FET, frozen embryo transfer; OPU, oocyte pickup.

In all cycles, 65.8% (*n* = 9,656) of patients were ≤34 years at OPU and 59.7% (*n* = 8,766) were ≤34 years at warm. For FET cycles with embryos cryopreserved >5 years, 78.5% (*n* = 223) of patients were ≤34 years at OPU and 46.1% (*n* = 131) were 35–39 years at warm.

#### Outcomes in relation to parity

Table 3 displays OPR and LBR stratified by parity for all FET cycles and cycles with embryos cryopreserved >5 years. It also includes the statistical analysis of OPR and LBR between patients receiving treatment for the first vs second child and first vs third/fourth child for all FET cycles and cycles with embryos cryopreserved >5 years. For comparisons of OPR and LBR in combination with patients’ relationship status, see Supplementary Table 3.

**Table 3:** OPR and LBR for all FET cycles and FET cycles with embryos cryopreserved >5 years by parity status. Boldface was used to highlight statistical significance, *P* < 0.05. Patients who had multiple cycles with warm are represented more than once.

Duration of cryopreservation	Parity, FET for			
	1st child	2nd child	3rd/4th child*	All
All FET cycles				
Cycles with warm	11,538	3,014	125	14,677
Cycles with transfer	11,370	2,940	123	14,433
OPR per transfer	36.7% (4,174/11,370)	42.6% (1,253/2,940)	48.8% (60/123)	38.0% (5,487/14,433)
*P*-value	-	**<0.01^†^**	**0.01^†^**	-
Odds ratio (95% CI)	-	1.26 (1.16–1.38)	1.63 (1.11–2.38)	-
LBR per transfer	31.3% (3,556/11,370)	35.6% (1,046/2,940)	39.0% (48/123)	32.2% (4,650/14,433)
*P*-value	-	**<0.01^†^**	0.1^†^	-
Odds ratio (95% CI)	-	1.19 (1.08–1.30)	1.39 (0.94–2.05)	-
FET cycles (>5 years)				
Cycles with warm	64	159	61	284
Cycles with transfer	60	152	60	272
OPR per transfer	31.7% (19/60)	34.9% (53/152)	45.0% (27/60)	36.4% (99/272)
*P*-value	-	0.60^‡^	0.15^‡^	-
Odds ratio (95% CI)	-	1.21 (0.60–2.45)	1.9 (0.80–4.49)	-
LBR per transfer	25.0% (15/60)	30.3% (46/152)	38.3% (23/60)	30.9% (84/272)
*P*-value	-	0.40	0.14	-
Odds ratio (95% CI)	-	1.43 (0.64–3.22)	2.10 (0.79–5.63)	-

* Only one FET cycle was performed for a patient/couple’s 4th child.

^†^ The *P*-value was calculated in reference to FET cycles for first child (all FET cycles).

^‡^ The *P*-value was calculated in reference to FET cycles for first child (FET cycles ≥5 years).

OPR, ongoing pregnancy rate. Heartbeat confirmed with ultrasound in gestational week 7–8. LBR, live birth rate; FET, frozen embryo transfer.

Among all FET cycles, the OPR and LBR were, respectively, 36.7 and 31.1% for patients receiving treatment for the first child, 42.6 and 35.6% for the second child, and 48.8 and 39.0% for the third/fourth child. Statistically significant differences in OPR and LBR were found in FET for the first vs second child (*P* < 0.01; OR = 1.26, 95% CI: 1.16; 1.38 and *P* < 0.01; OR = 1.19, 95% CI: 1.08; 1.30), indicating higher odds of obtaining an ongoing pregnancy and live birth for the second child compared with the first child. In the same manner, a significant difference was also found in OPR in patients receiving treatment for the third/fourth child vs first child (*P* = 0.01; OR = 1.63; 95% CI: 1.11; 2.38), but LBR did not reach statistical significance.

For FET cycles with embryos cryopreserved >5 years, OPR and LBR were, respectively, 31.7 and 25.0% for patients receiving treatment for the first child, 34.9 and 30.3% for the second child, and 45.0 and 38.3% for the third/fourth child. There was no significant difference in OPR or LBR between the three parity groups.

#### Outcomes in relation to storage time

Table 4 includes a subgroup analysis comparing OPR and LBR in FET cycles with embryos cryopreserved ≤5 years vs >5 years. Across parity groups, no significant differences in OPR or LBR were observed between FET cycles with embryos cryopreserved ≤5 years vs >5 years. Similarly, no statistically significant differences in OPR or LBR were found when comparing all FET cycles with embryos cryopreserved ≤5 years vs >5 years.

**Table 4:** Comparison of OPR and LBR by parity for FET cycles with embryos cryopreserved ≤5 years vs >5 years. Patients who had multiple cycles with warm are represented more than once.

Parity	Duration of cryopreservation		*P*-value	Odds ratio (95% CI)
	≤5 years	>5 years		
FET for first child				
Cycles with warm	11,474	64	-	-
Cycles with transfer	11,310	60	-	-
OPR per transfer	36.7% (4,154/11,310)	31.7% (19/60)	0.39	0.78 (0.44–1.38)
LBR per transfer	31.3% (3,541/11,310)	25.0% (15/60)	0.26	0.70 (0.40–1.30)
FET for second child				
Cycles with warm	2,855	159	-	-
Cycles with transfer	2,788	152	-	-
OPR per transfer	43.0% (1,200/2,788)	34.9% (53/152)	0.05	0.70 (0.48–1.01)
LBR per transfer	35.9% (1,000/2,788)	30.3% (46/152)	0.18	0.77 (0.52–1.13)
FET for third/fourth child				
Cycles with warm	64	61	-	-
Cycles with transfer	63	60	-	-
OPR per transfer	52.4% (33/63)	45.0% (27/60)	0.43	0.74 (0.35–1.56)
LBR per transfer	39.7% (25/63)	38.3% (23/60)	0.87	0.94 (0.43–2.03)
All FET cycles				
Cycles with warm	14,393	284	-	-
Cycles with transfer	14,161	272	-	-
OPR per transfer	38.0% (5,387/14,161)	36.4% (99/272)	0.50	0.91 (0.70–1.19)
LBR per transfer	32.2% (4,566/14,161)	30.9% (84/272)	0.54	0.92 (0.70–1.21)

FET, frozen embryo transfer; LBR, live birth rate; OPR, ongoing pregnancy rate. Heartbeat confirmed with ultrasound in gestational week 7–8.

## Discussion

As of March 1, 2024, a total of 29,591 cryopreserved embryos were in storage in the public fertility clinics in Denmark. Between January 1, 2017, and December 31, 2019, an average of 1,399 embryos was destroyed annually because they reached the maximum storage period of five years. In this study, FET cycles performed after the legislative amendment allowing embryo cryopreservation until the woman reaches 46 years were analyzed (*n* = 14,677). FET cycles with embryos cryopreserved ≤5 years represented 98.1% (*n* = 14,393) of all included cycles, and only 1.9% (*n* = 284) of FET cycles involved embryos cryopreserved >5 years. Among all FET cycles, OPR and LBR increased with increasing parity, although LBR for the first vs third/fourth child did not reach statistical significance. No statistically significant differences in OPR or LBR were observed with increasing parity in FET cycles with embryos cryopreserved >5 years. When comparing all FET cycles and parity groups with embryos cryopreserved ≤5 years vs >5 years, no significant differences were found in OPR or LBR. Among FET cycles with embryos cryopreserved >5 years, 99 cycles resulted in an ongoing pregnancy and 84 resulted in a live birth.

To the best of our knowledge, no other studies have reported on the use of embryos in Denmark following the legislative amendment extending the maximum duration of embryo cryopreservation. A Danish study has recently been published on the outcomes in terms of implantation and live birth rates for FET cycles with blastocysts with a Gardner score ≥2BB based on storage time, reporting that blastocysts cryopreserved >25 months did not significantly affect pregnancy or live birth chances (Veng Eskildsen *et al.* 2025). Several international studies have also investigated the effects of prolonged embryo cryopreservation on post-warming survival and clinical outcomes, but the studies provide contradictory findings and use varying definitions for prolonged cryopreservation. Few studies have assessed clinical outcomes of FET with embryos cryopreserved >5 years and those that have typically involve small sample sizes of FET cycles with prolonged embryo storage duration >5 years (Pruksananonda *et al.* 2012, Ueno *et al.* 2018, Ma *et al.* 2021, Li *et al.* 2023, Yan *et al.* 2023, Zhan *et al.* 2024).

Several studies have reported that transfer of embryos after prolonged cryopreservation duration does not adversely affect clinical outcomes. Ueno *et al.* conducted a retrospective, single-center cohort study on 8,736 vitrified–warmed blastocyst transfer cycles, with a maximum storage duration of eight years, and found that cryopreservation duration did not affect LBR (Ueno *et al.* 2018). Similarly, Li *et al.* found that pregnancy and neonatal outcomes after transfer of vitrified blastocysts and cleavage-stage embryos were not impaired by cryopreservation up to seven years, and Ma *et al.* found similar results (Ma *et al.* 2021). Canosa *et al.* defined prolonged storage of vitrified blastocysts and cleavage-stage embryos as >12 months and found that survival, miscarriage, live birth, and major malformation rates were similar between FET cycles with embryos cryopreserved >12 months and those stored for shorter periods, but they did not adjust for potential confounders (Canosa *et al.* 2022). When attempting to derive embryonic stem cells, Pruksananonda *et al.* demonstrated that the pluripotency of human embryos cryopreserved for up to 18 years was comparable to fresh embryos and found no apparent adverse effects of long-term storage on the developmental potential of the embryo (Pruksananonda *et al.* 2012).

On the contrary, other studies have reported more negative associations of prolonged cryopreservation duration of embryos on implantation, pregnancy, and live birth rates. Mao *et al.* observed in their retrospective cohort study on vitrified–warmed embryos from 31,143 patients that embryo survival rate significantly decreased with longer cryopreservation duration. Long-term storage was defined as >2 years, and clinical pregnancy rates evidently decreased with storage time over three months. Neonatal health was not significantly affected by prolonged cryopreservation, and Mao *et al.* deemed FET with long-term cryopreserved embryos as clinically feasible (Mao *et al.* 2022). Similarly, Yan *et al.* analyzed FET cycles with vitrified blastocysts cryopreserved up to 10.5 years and found that long-term cryopreservation over six years had a negative effect on biochemical pregnancy, clinical pregnancy, and LBR, but did not have a significant impact on neonatal outcomes (Yan *et al.* 2023). Zhan *et al.* reported that prolonged cryopreservation of vitrified cleavage-stage embryos did not affect implantation rate and LBR, but good-quality vitrified blastocysts cryopreserved >5 years were associated with lower implantation and live birth rates (Zhan *et al.* 2024).

In this study, no statistically significant differences in OPR or LBR were observed between FET cycles with embryos cryopreserved ≤5 years vs >5 years. Among FET cycles with embryos cryopreserved >5 years, the overall OPR and LBR were 36.4 and 30.9%, respectively, comparable to the clinical pregnancy rate of 36.0% reported by the Danish Fertility Society (DFS) in 2025 for FET cycles with embryo transfer across public and private clinics in Denmark (Danish Fertility Society 2025, 2026).

Although patients with FET cycles with embryos cryopreserved >5 years constitute a small subgroup in this study, the findings suggest that the legislative amendment permitting prolonged cryopreservation has enabled patients to make longer-term reproductive plans. Particularly for those seeking additional pregnancies following an initial FET success, the amendment has provided the opportunity to use surplus embryos, even if FET is delayed beyond five years from the initial oocyte pickup. Furthermore, prior to the legislative amendment, 4,196 embryos were destroyed over a three-year period due to the five-year storage limit, suggesting that some patients may have had embryos destroyed that could have been used for future family expansion. Supporting this, a study by Bangsbøll *et al.* on 284 Danish couples who had experienced destruction of their cryopreserved embryos because of the then maximum storage limit of two years found that 59% of respondents cited ‘too short legislative limit for cryopreservation’ as a reason for not using their surplus embryos (Bangsbøll *et al.* 2004).

When analyzing all FET cycles by parity status, a statistically significant increase in OPR for patients receiving treatment for the second or third/fourth child compared with the first child was found, as well as a significant increase in LBR for the second child compared with the first child. In line with this, Lawrenz *et al.* found that OPR was significantly higher among patients who had previously achieved at least one pregnancy, which could be explained by a ‘functioning’ reproductive system in the prospective mother (Lawrenz *et al.* 2024). The same significant increase in OPR or LBR with increasing parity was not found in FET cycles with embryos cryopreserved >5 years, maybe due to the small sample size.

### Strengths and limitations

A strength of this study is the inclusion of nationwide data from all the public fertility clinics in Denmark and thereby the large sample size. For the primary clinical outcome, ongoing pregnancy was assessed. Live birth was included in the analysis but was not the primary clinical outcome due to missing data on live birth outcome, accounting for 3.6% (*n* = 533) of cycles. Other limitations include the absence of precise data on patient age at OPU. Consequently, age at OPU was not adjusted for as a potential confounder in the analysis of OPR and LBR. The post-warming survival rate was estimated as the number of embryos transferred divided by the total number of embryos warmed. However, the absence of a transfer does not necessarily indicate reduced viability following warming. Finally, the subgroup analysis was limited by the small number of FET cycles with embryos cryopreserved >5 years.

## Conclusion

This study provides insights into the embryo usage, OPR, and LBR for FET cycles after the introduction of the Danish legislation allowing prolonged cryopreservation until the woman turns 46 years. The study found that in a three-year period before the legislative amendment, an average of 1,399 embryos was destroyed annually because they reached the maximum storage time of five years, suggesting that patients may have had embryos destroyed that could have been used for FET.

Overall, the study showed comparable clinical outcomes in terms of OPR and LBR for FET cycles with embryos cryopreserved ≤5 years vs >5 years and showed higher OPR and LBR with increasing parity (LBR for the first vs third/fourth child did not reach statistical significance). In addition, 20.5% (*n* = 3,014) of all patients received treatment for the second child compared with 56.0% (*n* = 159) of patients with FET with embryos cryopreserved >5 years. During the study period, 84 children were born from embryos cryopreserved >5 years that previously would have been destroyed. These findings indicate that the extension of the maximum duration of embryo cryopreservation may have enabled patients to use their surplus embryos for family expansion.

A follow-up study in a few years is recommended, as the number of patients who have used embryos cryopreserved >5 years is likely to have increased. This study provides a descriptive overview but does not allow for definitive conclusions regarding the clinical impact of prolonged embryo cryopreservation. The heterogeneity in the existing literature highlights the need for further research to clarify the effect and safety of extended cryopreservation duration on clinical outcomes.

## Supplementary materials



## Declaration of interest

The authors declare that there is no conflict of interest that could be perceived as prejudicing the impartiality of the work reported.

## Funding

This research did not receive any specific grant from funding agencies in the public, commercial, or not-for-profit sectors.

## Author contribution statement

EHP contributed to data acquisition and data analysis and drafted the manuscript. AZ conceptualized and designed the study and contributed to data acquisition and revising the manuscript. MRP, MLG, AG, SRH, TVE, BBP, and BT contributed to data acquisition and revising the manuscript. DFS performed the data analysis. ESD, HSN, and NLCF supervised the project and revised the manuscript.

## Ethical approval

The data from the eight clinics were obtained from the Danish Medical Data Center (DMDC). The use of registry and journal data was approved by the Secretary for Health Law under journal number R-24012183, in accordance with section 46, subsection 2 of the Danish Health Act (Sundhedsloven § 46, stk. 2). Approval was granted on March 24, 2024.
