# Supplementary material for: Effects of prolonged embryo cryopreservation after fertility treatment in Denmark
Source: Reprod Fertil. 2026 Jun 18;7(2):RAF250185. doi: 10.1530/RAF-25-0185 (PMC13280622; doi:10.1530/RAF-25-0185)
Supplement: Supplementary file 1 [file supplementary_materials.pdf]

Supplementary Table 1- local vitrification procedures at all clinics

Clinic	Number of embryos warmed	Vitrification Device	Vitrification/Warming Media	Assisted blastocyst collapse before vitrification
A	2,072	Rapid-i (Vitrolife)	Vit Kit- Freeze /Warm (IrvineScientific)<br>Vit Kit-freeze NX / Warm NX (IrvineScientific)	No
B	2,204	Rapid-i (Vitrolife)	Vit Kit- Freeze / Warm (IrvineScientific)<br>Vit Kit-freeze NX / Warm NX (IrvineScientific)	Yes
C	2,048	Rapid-i (Vitrolife)	Vit Kit- Freeze / Warm (IrvineScientific)<br>Vit Kit-freeze NX / Warm NX (IrvineScientific)	Yes
D	1,346	Rapid-i (Vitrolife)	Vit Kit- Freeze / Warm (IrvineScientific)<br>Vit Kit-freeze NX / Warm NX (IrvineScientific)	No
E	888	Rapid-i (Vitrolife)	RapidVit and RapidWarm omni (Vitrolife)	No
F	2,644	Rapid-i (Vitrolife)	RapidVit and RapidWarm omni (Vitrolife)	Yes
G	2,260	Cryotech (Cryotech)	Vitrification and warming media (KitaZato)	No
H	1,730	Cryotop (Kitazato)<br>Cryotech (Cryotech)	Vitrification and warming media (KitaZato)<br>Vit Kit- Freeze / Warm (IrvineScientific)<br>Vit Kit-freeze NX / Warm NX(IrvineScientific)	No

Supplementary Table 2- Age distribution at OPU and embryo warming by patient group

Uxor age<br><br>Patient Group			≤34 years	35-39 years	40-42 years	≥43 years
Heterosexual couples using partner's sperm	All FET Cycles	Age at OPU	71.4% (8,870/12,431)	23.4% (2,904/12,431)	5.2% (648/12,431)	0.1% (9/12,431)
		Age at warm	64.8% (8,056/12,431)	27.8% (3,456/12,431)	6.7% (832/12,431)	0.7% (87/12,431)
	FET Cycles (> 5 years)	Age at OPU	82.9% (204/246)	15.9% (39/246)	1.2% (3/246)	-
		Age at warm	32.9% (81/246)	47.6% (117/246)	11.8% (29/246)	7.7% (19/246)
Heterosexual couples using donor sperm	All FET Cycles	Age at OPU	64.3% (241/375)	29.1% (109/375)	6.7% (25/375)	-
		Age at warm	60.8% (228/375)	32.0% (120/375)	7.2% (27/375)	-
	FET Cycles (> 5 years)	Age at OPU	100% (2/2)	-	-	-
		Age at warm	-	100% (2/2)	-	-
Single women and lesbian couples using donor sperm	All FET Cycles	Age at OPU	29.8% (518/1,739)	46.8% (813/1,739)	23.5% (408/1,739)	-
		Age at warm	26.4% (459/1,739)	44.7% (778/1,739)	26.7% (464/1,739)	2.2% (38/1,739)
	FET Cycles (> 5 years)	Age at OPU	53.3% (16/30)	33.3% (10/30)	13.3% (4/30)	-
		Age at warm	16.7% (5/30)	36.7% (11/30)	16.7% (5/30)	30.0% (9/30)
Unknown relationship status	All FET Cycles	Age at OPU	20.5% (27/132)	50.8% (67/132)	28.8% (38/132)	-
		Age at warm	17.4% (23/132)	38.6% (51/132)	42.4% (56/132)	1.5% (2/132)
	FET Cycles (> 5 years)	Age at OPU	16.7% (1/6)	50.0% (3/6)	33.3% (2/6)	-
		Age at warm	-	16.7% (1/6)	33.3% (2/6)	50.0% (3/6)

Uxor ages from all FET cycles (n=14,677) and from FET cycles with embryos cryopreserved > 5 years (n=284) are included. Patients who had multiple cycles with warm are therefore represented more than once.

Supplementary Table 3 – OPR and LBR by patient group and parity status

Parity Group<br><br>Patient Group			FET for first child	FET for second child	FET for third/fourth [a]	FET all parity
Heterosexual couples using partner's sperm	All FET cycles	Cycles with warm	9,680	2,636	115	12,431
		Cycles with transfer	9,545	2,574	114	12,233
		OPR per transfer	37.4% (3,566/9,545)	43.5% (1,120/2,574)	50.0% (57/114)	38.8% (4,743/12,233)
		LBR per transfer	32.1% (3,060/9,545)	36.1% (929/2,574)	39.5% (45/114)	33.0% (4,034/12,233)
	FET Cycles (> 5 years)	Cycles with warm	59	128	59	246
		Cycles with transfer	55	125	58	238
		OPR per transfer	34.6% (19/55)	38.0% (49/125)	44.8% (26/58)	39.5% (94/238)
		LBR per transfer	27.3% (15/55)	34.4% (43/125)	37.9% (22/58)	33.6% (80/238)
Heterosexual couples using donor sperm	All FET cycles	Cycles with warm	308	67	0	375
		Cycles with transfer	308	67	0	375
		OPR per transfer	33.1% (102/308)	46.3% (31/67)	-	35.5% (133/375)
		LBR per transfer	28.9% (89/308)	38.8% (26/67)	-	30.7% (115/375)
	FET Cycles (> 5 years)	Cycles with warm	1	1	0	2
		Cycles with transfer	1	1	0	2
		OPR per transfer	0% (0/1)	100% (1/1)	-	50.0% (1/2)
		LBR per transfer				
Single women and lesbian couples using donor sperm	All FET cycles	Cycles with warm	1,442	288	9	1,739
		Cycles with transfer	1,442	288	9	1,739
		OPR per transfer	33.4% (481/1,442)	34.4% (99/288)	33.3% (3/9)	33.5% (583/1,739)
		LBR per transfer	27.0% (390/1,442)	30.6% (88/288)	33.3% (3/9)	27.7% (481/1,739)
	FET Cycles (> 5	Cycles with warm	3	25	2	30

	years)	Cycles with transfer	3	25	2	30
		OPR per transfer	0% (0/3)	12.0% (3/25)	50.0% (1/2)	13.3% (4/30)
		LBR per transfer	0% (0/3)	8.0% (2/25)	50.0 (1/2)	10.0% (3/30)
Unknown relationship status	All FET cycles	Cycles with warm	108	23	1	132
		Cycles with transfer	75	11	0	86
		OPR per transfer	33.3% (25/75)	27.3% (3/11)	-	32.6% (28/86)
		LBR per transfer	22.7% (17/75)	27.3% (3/11)	-	23.3% (20/86)
	FET Cycles (> 5 years)	Cycles with warm	1	5	0	6
		Cycles with transfer	1	1	0	2
		OPR per transfer	0% (0/1)	0% (0/1)	-	0% (0/2)
		LBR per transfer	0% (0/1)	0% (0/1)	-	0% (0/2)

a. Only one FET cycle was performed for a patient/couple's 4th child

Patients who had multiple cycles with warm are represented more than once

OPR: Ongoing pregnancy rate. Heartbeat confirmed with ultrasound gestational week 7-8

LBR: Live birth rate
